# Differential Expression of CD45RO and CD45RA in Bovine T Cells

**DOI:** 10.3390/cells11111844

**Published:** 2022-06-04

**Authors:** Anmol Kandel, Lei Li, Akanksha Hada, Zhengguo Xiao

**Affiliations:** Department of Animal and Avian Sciences, University of Maryland, College Park, MD 20742, USA; akandel1@umd.edu (A.K.); lixxx242@umd.edu (L.L.); hada@umd.edu (A.H.)

**Keywords:** cattle, memory, CD4+ T cells, CD8+ T cells, γδ T cells, CD45RO, CD45RA, IFNγ, IL4, PBMCs

## Abstract

Effective vaccination induces immune memory to protect animals upon pathogen re-encounter. Despite contradictory reports, bovine memory T cells are identified based on two isoforms of CD45, expression of CD45RO plus exclusion of CD45RA. In this report, we contrasted CD45RA/RO expression on circulatory T cells with IFNγ and IL4 expression induced by a conventional method. To our surprise, 20% of cattle from an enclosed herd did not express CD45RO on T cells without any significant difference on CD45RA expression and IFNγ or IL4 induction. In CD45RO expressing cattle, CD45RA and CD45RO expressions excluded each other, with dominant CD45RO (>90%) expression on gamma delta (γδ) followed by CD4+ (60%) but significantly higher CD45RA expression on CD8+ T cells (about 80%). Importantly, more than 80% of CD45RO expressing CD4+ and CD8+ T cells failed to produce IFNγ and IL-4; however, within the cytokine inducing cells, CD4+ T cells highly expressed CD45RO but those within CD8+ T cells mostly expressed CD45RA. Hence, CD45RO is not ubiquitously expressed in cattle, and rather than with memory phenotype, CD45RA/RO expression are more associated with distinct T cell subtypes.

## 1. Introduction

Memory T cells mount rapid and robust immune responses and are the hallmark of effective vaccination. The quality and quantity of induced immune memory are therefore critical for evaluating the efficacy of vaccines. Conventionally, memory T cells were detected as cells producing cytokines such as IFNγ and IL4 after brief stimulation in vitro [1,2,3]. However, more recently, memory T cells are often identified based on the inclusion of CD45RO and exclusion of CD45RA on T cells, which is a relatively easy and practical approach for downstream treatments [3,4,5]. In humans and bovine T cells, CD45RA and CD45RO expressions are found to be mutually exclusive, while naïve cells are defined as CD45RA positive (CD45RA+) and CD45RO negative (CD45RO-), memory T cells are CD45RO positive (CD45RO+) and CD45RA negative (CD45RA-) [6,7,8]. The small population of double positive (DP) cells that co-express CD45RA and CD45RO (i.e., CD45RA+/CD45RO+) are believed to reflect the transition stage from naïve to memory induced by pathogen infection or vaccination [9,10,11]. Despite some controversial reports, the conventional memory markers CD45RO and CD45RA are used extensively to detect memory T cells in humans and cattle [5,12,13,14,15,16,17,18,19,20,21,22,23,24,25].

CD45 is a tyrosine phosphatase membrane protein expressed abundantly on the surface of a wide range of immune cells, including T lymphocytes [26,27]. In humans, CD45 is expressed in a combination of multiple isoforms generated through alternative splicing of the CD45 pre-mRNA at exons 4, 5, and 6 [26,28,29]. Inclusion and exclusion of these three exons differentially generate CD45 isoforms, such as the high molecular weight CD45RA and the low molecular weight CD45RO [26,30]. Specifically, the shortest CD45RO transcript is generated through the exclusion of exons (4, 5, and 6) while still retaining 3 and 7, which are shared by all the other isoforms [26,30]. Therefore, selective detection of CD45RO transcript by quantitative PCR (qPCR) is not practical. Instead, due to the highly conserved nature of CD45 protein across the mammalian species [28,31], bovine CD45RO expression is commonly examined at the protein level using a monoclonal antibody (clone # ILA116) in flow cytometry [5,8,24,30,32,33,34,35,36,37,38]. Bembridge et al. (1995) in their seminal research first used this monoclonal antibody (clone #ILA116) to precipitate bovine CD45RO, which had a molecular weight very close to that of humans (180 kDa), and suggested that this isoform could be associated with memory [8]. Subsequently, Sopp et al. (2005) reported that CD45RO+ CD4+ T cells were the dominant producer of IFNγ and IL4 [23]. These reports, along with several others, supported the idea that CD45RO expression is associated with memory phenotype in cattle [8,23,24,39].

In the past few decades, a considerable number of reports in humans and cattle challenged the classical CD45RA/RO paradigm that defines the memory phenotype as CD45RO+ and CD45RA- cells. Specifically, a large number of reports in humans reveal the existence of terminally differentiated CD45RA+ memory T cells both in vitro and in vivo, indicating that memory T cells could be found within both CD45RO+ and CD45RA+ subpopulations [13,16,18,19,21,22]. Interestingly, some data in bovine research also contrast this classical paradigm. For instance, Hagberg et al. (2008) suggested that CD45RO+ CD4+ and CD8+ T cells isolated from immune cattle failed to proliferate in response to the homogenate derived from the *Dictyocaulus viviparous* parasite [40,41]. Further, memory CD8+ T cells were detected in both CD45RO+ and CD45RA+ fractions, suggesting that CD45RO is not necessarily a memory marker for bovine CD8+ T cells [8,9,37,42,43]. In addition, Guerra Maupome et al. (2019) suggested that CD45RO is not an appropriate marker to detect bovine memory γδ T cells [44]. These contradictory reports warrant re-assessment of the classical paradigm in cattle to verify if CD45RA/RO isoforms are genuinely associated with memory phenotype in bovine T cells.

This project aimed to contrast the CD45RA/RO expression on circulatory T cells with memory T cell subtypes from the same cattle. Our data suggest that the classical CD45RA/RO paradigm may not be accurate in cattle. First, about 20% of cattle (six out of 28) did not express CD45RO. Furthermore, in those expressing CD45RO, the expression of this isoform was not correlated with IFNγ and IL4 induction. Instead, CD45RO and CD45RA clustered differentially in bovine T cell subtypes, suggesting that their expression is more associated with T cell subtypes than with memory phenotype.

## 2. Materials and Methods

### 2.1. Cattle

A total of 28 healthy cattle (Wye Angus), including 20 grass-fed and eight grain-fed, aged from one year to 24 months, were used. The closed herd was housed at the Wye Research and Education Center, University of Maryland Experimental Station (Queenstown, MD, USA) [45,46,47,48]. All calves were weaned at six months of age, then randomly assigned to either grain-fed or grass-fed groups. The grain-fed group was kept on a feedlot with a mixture diet [46], and the grass-fed group was maintained on the pasture as reported previously [49]. All the animals were examined monthly to ensure health. Animal Care and Use Protocols were approved by UMD (R-FEB-18-06 and R-JAN-21-02), Institutional Animal Care and Use Committee (IACUC), with the relevant guidelines and regulations.

### 2.2. Peripheral Blood Mononuclear Cells (PBMCs) Isolation and Stimulation

This procedure was similarly performed as in our previous reports [47,48]. Briefly, fresh blood was collected from the jugular vein using EDTA coated vacutainers (Becton Dickinson Vacutainer Systems, Franklin Lakes, NJ, USA) and transferred to 15 mL conical tubes (Fisher Scientific, Pittsburgh, PA, USA), which were centrifuged for 30 min at 1200× *g*. Following centrifugation, the buffy coat at the interface was gently collected into a new 15 mL tube and re-suspended with 1× phosphate-buffered saline (PBS) (Fisher Scientific, Fair Lawn, NJ, USA) to 8 mL. Then, it was overlaid with a 5 mL lymphocyte separation medium (LSM) with the density of 1.077 g/mL (Corning, Manassas, VA, USA), followed by centrifugation for 30 min at 900× *g* with break off. The second buffy coat at the interface was collected and washed twice with PBS. After the last wash, the cell pellet was re-suspended in 5 mL Allos medium (RPMI1640 (Corning, Manassas, VA, USA) plus 10% FBS), and a small aliquot was used for cell counting.

Following the purification, one million cells per sample were aliquoted and re-suspended in 1 mL Allos medium supplied with Brefeldin A (BFA) (BioLegend, San Diego, CA, USA) or cell activation cocktail (CT) (R&D Systems, Minneapolis, MN, USA) that contains monensin sodium salt (1.5 mM), Phorbol 12-myristate 13-acetate (0.0405 mM) and Ionomycin calcium salt (0.67 mM). The cell suspensions were incubated at 37 °C with 5% CO_2_ for 4 h before intracellular staining.

### 2.3. Antibodies and Reagents

All the antibodies used in this study are listed in the following tables: primary antibodies (Table 1) and secondary antibodies plus isotypes (Table 2). Staining buffer (SB) is PBS with 2% FBS, and fix solution is 4% paraformaldehyde (W/V) in PBS with pH 7.4. Intracellular staining permeabilization wash buffer (P/W) (BioLegend, San Diego, CA USA) was purchased and used following the manufacturer instructions.

### 2.4. Flow Cytometry Analysis

For surface staining, cells were stained with primary antibodies (Table 1), followed by secondary antibodies, or fluorescence conjugated antibodies (Table 2) at 4 °C for 25 min, then washed with SB to remove the unbounded antibodies. Cells were further incubated with fix solution (1:1 dilution) for 15 min and washed with SB. Finally, cells were re-suspended in SB before being analyzed in a flow cytometer. For intracellular staining, the cells were stimulated for 4 h as described in 2.2, and then fixed with 2% paraformaldehyde for 15 min. Following two washes, cells were incubated with permeabilization wash buffer (P/W) (BioLegend, San Diego, CA, USA) for additional 15 min at 4 °C. Similar to the surface staining, cells were incubated with antibodies at 4 °C for 25 min. Finally, cells were re-suspended in SB for reading. Staining of isotype and unstained controls was performed using the same method described above. Cells were examined by using FACSCalibur flow cytometer. The Flow cytometer data were analyzed with the FlowJo version 10 (Tree Star, Ashland, OR, USA).

### 2.5. Statistical Analysis

Statistical analyses were performed using Prism 9 (GraphPad Software, Inc., La Jolla, CA, USA), and details are described in the figure legends. Overall, all data passed the Anderson–Darling normality test and were analyzed via one-way ANOVA with Tukey’s Multiple Comparisons Test. Asterisks indicate statistical significance. * *p* < 0.05; ** *p* < 0.01; *** *p* < 0.001; **** p < 0.0001. NS indicates not significant.

## 3. Results

### 3.1. Bovine Lymphocytes Do Not Always Express CD45RO

**Figure 1:**
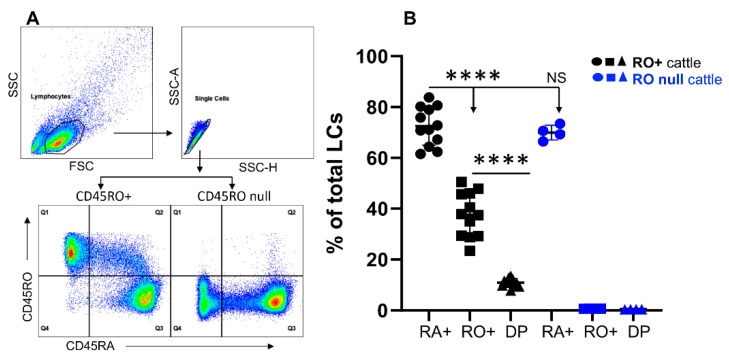
CD45RO is not expressed by lymphocytes in some cattle. Lymphocytes were gated in PBMCs and examined for expression of CD45RO and CD45RA. (**A**) Gating strategy for lymphocytes; (**B**) Comparison of CD45RO and CD45RA expression in lymphocytes. Data were pooled together from two experiments and expressed as mean of 12 CD45RO positive (**RO+**) cattle samples, or four CD45RO negative (**RO null**) cattle with standard deviation (SD). Data in BLUE will indicate samples from **RO null** cattle in the rest of figures. Q1: lymphocytes only expressing high level of CD45RO, but not CD45RA, designated as RO+; Q2: lymphocytes both CD45RO and CD45RA, designated as double positive (DP). Q3: lymphocytes only expressing high level of CD45RA, but not CD45RO, designated as RA+. Background in isotype control was subtracted from all the data in B. Data were analyzed by one-way ANOVA with Tukey’s Multiple Comparisons Test. Asterisks indicate statistical significance. **** * p* < 0.0001. NS: not significant. The same analysis will be performed in the rest of figures unless mentioned otherwise.

Detection of an established memory T cell population is the gold standard for evaluating effectiveness of the vaccines [35,50,51]. In both humans and cattle, memory cells are identified using CD45RO as a marker [23,25,31,52,53]. We examined CD45RO and CD45RA expressions in bovine PBMCs. Almost all of the lymphocytes expressed high levels of either CD45RO(RO+) or CD45RA(RA+), and a small population expressed both at intermediate levels, which are referred to as double positive (DP) cells throughout this manuscript (Figure 1A,B). In general, much more lymphocytes expressed CD45RA than CD45RO (Figure 1B). To our surprise, four out of sixteen cattle did not express CD45RO (designated as **RO null**) but their CD45RA expression level was similar to those in CD45RO expressing cattle, labeled as CD45RO positive **(RO+)** (Figure 1A,B). It seems lack of CD45RO isoform does not necessarily affect CD45RA expression. Among the examined cattle, while half of the cattle were raised on pasture (grass-fed), the other half were raised in feedlot (grain-fed) [46,48]. Due to more exposure of grass-fed cattle to the environmental pathogens present in the pasture such as *Ostertagia Ostertagi* (OO) [46], the frequency of the memory cells in these animals could be greater than in grain-fed counterparts, despite being under the same vaccination procedure [46,54]. The data were further analyzed by comparing the grass-fed cattle with the grain-fed, but no significant difference was observed (Supplementary Figure S1).

### 3.2. CD45RO and CD45RA Are Expressed Differentially in T Cell Subtypes

**Figure 2:**
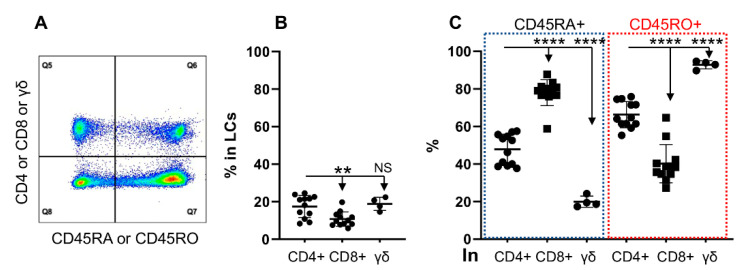
CD45RO and CD45RA are expressed differentially in T cell subtypes. Lymphocytes were gated in PBMCs and examined for the expression of CD45RO and CD45RA in different T cell subtypes. (**A**) Gating strategy for T cell subtypes and expression of CD45RO and CD45RA; (**B**) Comparison of frequency of T cell subtypes in PBMCs. (**C**) Comparison of CD45RO and CD45RA expressions in CD4+, CD8+ and γδ T cells. The same samples were examined as in Figure 1. Blue dotted box: comparison of CD45RA+% among different T cells subtypes; Red dotted box: comparison of CD45RO+% among different T cells subtypes. Q5: T cell subpopulation not expressing CD45RA or RO. Q6: T cell subpopulation expressing CD45RA or RO. Percentage of CD45RA+ or CD45RO+ in T cell subtype was expressed as Q6/(Q5 + Q6), with background subtracted using isotype control. ** *p* < 0.01, **** *p* < 0.0001.

There are three major T cell subtypes in cattle, CD4+, CD8+ and gamma delta (γδ) T cells, all of which could differentiate into memory cells, with CD45RO as the marker [8,23,55]. Consistent with previous literature, CD4+ and γδ were the biggest T cell populations in PBMCs, while the CD8+ population was significantly lower than that of CD4+ T cells (Figure 2B) [23,56]. Interestingly, CD45RO and CD45RA expression clustered differently in each T cell subtype with γδ T cells (90%) dominantly expressing CD45RO, followed by CD4+ (around 60%), and CD8+ (around 40%) as shown in Figure 2C. The pattern of CD45RA expression, as expected [8,44], was opposite to CD45RO, lowest in γδ T cells but highest in CD8+ T cells (Figure 2C). It seems that the expression of CD45RA and CD45RO is associated with specific T cell subtypes. With the reason unknown, CD45RO expression was higher in CD4+ from the grass-fed cattle than those from the grain-fed (Supplementary Figure S2B). 

### 3.3. Differential Activation Status in CD45RO+ and CD45RA+ Fractions of CD4+, but Not of CD8+ T Cells

**Figure 3:**
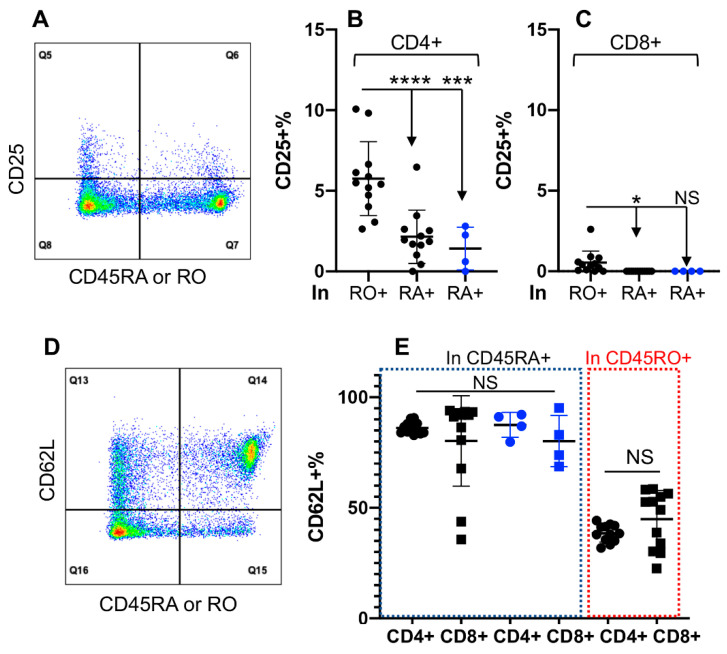
Activation status varies in CD45RO+ and CD45RA+ of CD4+ (**B**), but not of CD8+ T cells (**C**). CD45RO+ cells are designated as RO+ and CD45RA+ are labeled as RA+ in the figure (**B**,**C**). Lymphocytes were gated on CD4+ or CD8+ T cells in PBMCs and examined for the expression of CD25 and CD62L in either CD45RO+ or CD45RA+ subpopulations. (**A**,**D**) Gating strategies for CD25 (**A**) or CD62L (**D**) and expression of CD45RO and CD45RA based on gated CD4+ or CD8+ T cells. (**B**,**C**) Comparison of CD25+% in RO+ and RA+ subpopulations in CD4+ (**B**) and CD8+ (**C**) T cells. The same samples were analyzed as in Figure 1. Percentage of CD25+ expression in RO+ and RA+ subpopulations was expressed as Q6/(Q6 + Q7). (**E**) Comparison of CD62L+% in RO+ and RA+ subpopulations in CD4+ and CD8+ T cells. Percentage of CD62L+ expressing in CD45RO or CD45RA subpopulations was expressed as Q14/(Q14 + Q15). Background in isotype control was subtracted from all the data in (**B**,**D)**. * *p* < 0.05; *** *p* < 0.001; **** *p* < 0.0001.

CD25, the α subunit of IL-2 receptor, has been a reliable marker for identifying activated T cells in humans and cattle [48,57,58,59], which is also expressed by regulatory CD4+ T cells in cattle [46]. In the PBMCs from healthy cattle, only a small fraction of CD4+ T cells expressed CD25, thus potentially activated (Figure 3A,B). Majority of the cells in the CD25+ CD4+ T cell subpopulation were expressing CD45RO (RO+), with a smaller fraction expressing CD45RA (RA+) (Figure 3B). The frequency of RA+ cells in CD25+ CD4+ T cells was similar between **(RO+)** and **RO null** cattle (Figure 3B). In contrast, the frequency of CD25+ cells was significantly lower in CD8+ than in CD4+ T cells (*p* < 0.001) (Figure 3B,C and data not shown). CD62L, an adhesion molecule related to trafficking into the lymphoid tissues [60], was expressed at a higher level in CD45RA+ than in the CD45RO+ subpopulation from both CD4+ and CD8+ T cells (Figure 3D,E). The expression of CD62L was not affected by the lack of CD45RO expression in the **RO null** cattle (Figure 3E).

### 3.4. Expression of IFNγ and IL4 Is Not Associated with CD45RO in CD4+ T Cells

**Figure 4:**
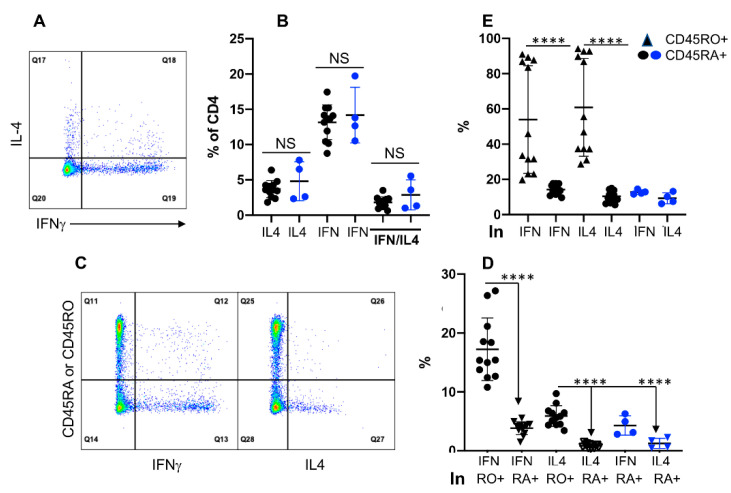
Expression of IFNγ and IL4 is not associated with CD45RO in CD4+ T cells. Purified PBMCs were stimulated with the activation cocktail for 4 h before intracellular staining for IFNγ and IL4, plus surface staining with antibodies to identify CD4+ T cells, and their expression of CD45RO and CD45RA. (**A**) Gating strategy for IFNγ and IL4 gated on CD4+ T cells. Q17: IL4+ subpopulation not expressing IFNγ, designated as IL4. Q18: IFNγ and IL4 double positive subpopulation, designated as **IFN/IL4**. Q19: IFNγ+ subpopulation not expressing IL4, designated as IFN. (**B**) Comparison of IFNγ and or IL4 producing CD4+ T cells between **RO+** and **RO null** cattle samples. (**C**) Gating strategies for IFNγ or IL4 producing CD4+ in CD45RO or CD45RA subpopulations. (**D**) Comparison of IFNγ or IL4 expression in RO+ or RA+ CD4+ T cells. Percentage of IFNγ+ was calculated as Q12/(Q11 + Q12), whereas IL4+ as Q26/(25 + 26). (**E**) Comparison of CD45RO+ or CD45RA+ in IFNγ+ or IL4+ CD4+ T cells. CD45RO+ or CD45RA+% in IFNγ+ was calculated as Q12/(Q12 + Q13), whereas in IL4+% as Q26/(26 + 27), as indicated in (**C**). Background in isotype control was subtracted from all the data in B, D, and E. **** *p* < 0.0001.

Induction of IFNγ and or IL4 after brief in vitro stimulation is a conventional method to detect memory T cells in humans and cattle [1,2,3,61]. Using this method, about 15% of the CD4+ T cells expressed IFNγ, whereas a smaller population expressed IL4, plus an even smaller fraction produced both IFNγ and IL4 (Figure 4A,B). Interestingly, the lack of CD45RO expression did not affect the frequencies of these IFNγ or IL4 producing cells (Figure 4B), suggesting CD45RO may not be an exclusive marker for memory CD4+ T cells. To further examine the association of IFNγ and/or IL4 with CD45RO, data were analyzed in two different ways. First, we calculated the percentages of cytokine producing cells within CD45RA+ (RA+) and CD45RO+ (RO+) subpopulations; second, we analyzed the frequencies of CD45RA and CD45RO within the cytokine producing cells. Close to 20% of the RO+ subpopulation produced IFNγ, which was much lower (about 5%) in RA+ (Figure 4D). This trend was similarly reflected in IL4 producing cells but at a lower level, and, and lack of CD45RO expression did not affect the frequency of IFNγ/IL4 production in **RO null** cattle (Figure 4D). In the second analysis, more than 50% of IFNγ or IL4 producing CD4+ T cells expressed CD45RO, with a significantly small fraction also expressing CD45RA (Figure 4E). There was no significant difference in the frequencies of IFNγ or IL4 producing CD4+ T cells between grass-fed and grain-fed cattle (Supplementary Figure S4A). Additionally, the percentage of CD45RO+ or CD45RA+ cells within IFNγ or IL4 producing populations was also similar (Supplementary Figure S4B). These data suggested that most cytokine-producing CD4+ T cells express CD45RO, consistent with previous reports in cattle [8,23,24,62], but a majority of the CD45RO expressing cells are not producing effector cytokines.

### 3.5. Expression of IFNγ Is Not Associated with CD45RO in CD8+ T Cells

**Figure 5:**
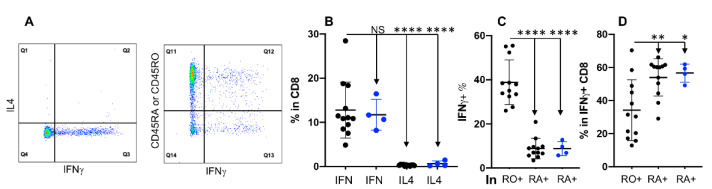
Expression of IFNγ is not associated with CD45RO in CD8+ T cells. The same samples and treatment were applied as in Figure 4, but the analysis was gated on CD8+ T cells. (**A**) Gating strategies for IFNγ+ and IL4+, plus IFNγ+ and CD45RO/CD45RA expression gated on CD8+ T cells. (**B**) Comparison between IFNγ (designated as IFN: Q2 + Q3) and IL4 (Q1 + Q2) producing cells in CD8. CD45RO+ cells were designated as RO+ and CD45RA+ cells as RA+ in (**C**,**D**). (**C**) Percentage of IFNγ+ in RO+ or RA+ CD8+ T cells: Q12/(Q11 + Q12), as indicated in (**A**). (**D**) Percentage of RO+ or RA+ in IFNγ producing CD8+ T cells: Q12/(Q12 + Q13), as indicated in (**A**). Background in isotype control was subtracted from all the date in B–D. * *p* < 0.05; ** *p* < 0.01; **** *p* < 0.0001.

Vaccination against intracellular pathogens such as viruses induces memory CD8+ T cells, which are critical for rapid control of infections [63,64]. We detected memory CD8+ cells using a similar methodology as applied for identifying memory CD4+ T cells in Figure 4. Unlike in CD4+, CD8+ T cells only produced IFNγ, but essentially no IL4 (Figure 5A,B). This is in agreement with their cytotoxic function instead of the regulatory function defined for CD4+ T cells that expressed both cytokines [64,65]. However, consistent with the results for CD4+ (Figure 4), the lack of CD45RO expression did not affect IFNγ production in CD8+ T cells from **RO null** cattle (Figure 5B). In CD8+ T cells, CD45RO expressing cells had a significantly higher frequency of IFNγ+ cells than those expressing CD45RA (Figure 5C), which was similar to observations in CD4+ T cells (Figure 4D). However, within IFNγ+ CD8+ T cells, the frequency of CD45RA expressing cells was significantly higher than those expressing CD45RO, opposite to the data in CD4+ T cells (Figure 4E), suggesting that CD45RA/RO expression was different between cytokine-producing CD4+ and CD8+ T cell subtypes. These data further support the notion that CD45RA/RO expression is associated with distinct T cell subtypes.

### 3.6. Expression of IFNγ and IL4 Is Not Associated with CD45RO in γδ T Cells

**Figure 6:**
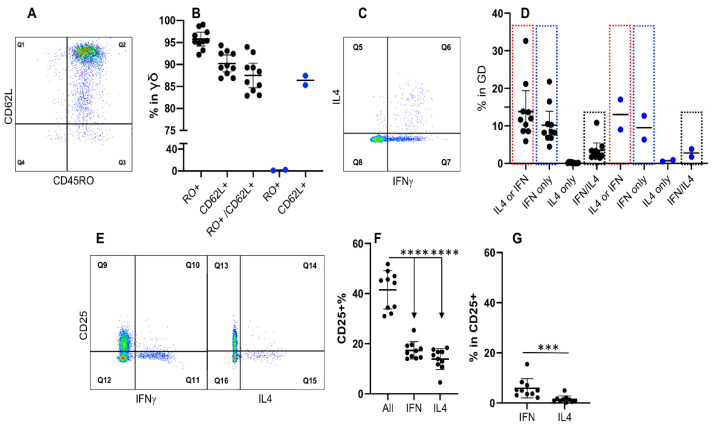
Expression of IFNγ and IL4 is not associated with CD45RO in γδ T cells. PBMCs were isolated from 12 cattle, which were tested for expression of CD45RO (RO+), CD62L (CD62L+), IFNγ (IFN) and IL4 in a way similar to that in Figure 4. (**A**) Gating strategy for RO+ and CD62L+ based on γδ T cells. (**B**) Comparison of CD62L expression in **RO+** cattle and **RO null** cattle. (**C**) Gating strategy for IFNγ and IL4+ gated on γδ T cells. (**D**) Comparison of IFNγ and IL4+ between **RO+** and **RO null** cattle. IL4 or IFN: Q5 + Q6 + Q7. Q5: IL4 only; Q6: IFN/IL4; Q7: IFN only. Red dotted box: IL4 or IFN; blue box: IFN only; black box: IFN/IL4. (**E**–**G**): Gating strategy for CD25+ and IFNγ+ or IL4+ based on γδ T cells (**E**), and comparison of their expression in both **RO+** and **RO null** cattle (**F**,**G**). In F, CD25+% in all: Q9 + Q10; CD25+% in IFN: Q10/(Q10 + Q11); CD25+% in IL4+: Q14/(Q14 + Q15). In G, IFN in CD25+: Q10/(Q9 + Q10); IL4 in CD25+: Q14/(Q13 + Q14). Data were expressed as mean plus 95% confidence interval (CI) in (**B**,**D**,**F**,**G**). Background in isotype control was subtracted from all the date in (**B**,**D**,**F**,**G**). *** *p* < 0.001; **** *p* < 0.0001.

γδ T cells have been suggested to be involved in both innate and adaptive immunity, thus their memory has also been identified with CD45RO in cattle [66,67]. Our data revealed that 2 out of 12 cattle were **RO null**, and, therefore, did not express CD45RO in the γδ T cells (Figure 6B and data not shown). In the **RO+** cattle, consistent with Figure 2, more than 90% of γδ T cells expressed CD45RO with majority of them also positive for CD62L (Figure 6A,B). Despite almost all of the γδ T cells (>90%) expressed CD45RO, only a small fraction of them produced IFNγ, suggesting weak or no association between CD45RO and memory phenotype (Figure 6C,D). Among the IFNγ expressing γδ T cells, a small frequency of cells was double positive for IFNγ/IL4, which was not affected by the lack of CD45RO expression in the **RO null** cattle (Figure 6C and dotted boxes in Figure 6D). Previously, γδ T cells have been reported to express low levels of CD25, [68,69,70], being activated at least partially. In our data, only a small fraction of IFNγ+ and IL4+ cells expressed CD25 (Figure 6E–G), suggesting that these cytokine expressing cells were mostly memory but not activated cells.

## 4. Discussion

Vaccine induces pathogen-specific memory T cells to protect animals from re-infection. Memory cells in cattle are defined as CD45RO+ and CD45RA- T cells. However, our research reveals that a fraction of cattle does not express CD45RO on T cells but still differentiate into IFNγ and IL4 producing memory cells. Additionally, in those that expressed CD45RO, the clustering of CD45RO and CD45RA isoforms is uniquely related to bovine T cell subtypes rather than the memory phenotype.

The classical paradigm supports that naïve cells are CD45RA+ CD45RO- and memory cells are CD45RO+ CD45RA-. However, some results from humans and cattle, at least partially, contrast this characterization [8,13,16,18,19,21,22,40,41,44]. In fact, multiple publications have suggested that the memory CD4+ and CD8+ T cells could express CD45RA. Therefore, the relevance of CD45RO as a unique memory marker for T cells remains controversial [12,13,14,15,16,17,18,19,20,21,22,71,72,73,74]. In support to these reports, our data demonstrated that, in a fraction (about 20%) of healthy cattle, T cells failed to express CD45RO but expressed a normal level of CD45RA. Interestingly, the frequency of IFNγ and IL4 producing memory cells in **RO null** cattle was not different from those in **RO+** cattle. This pattern was also consistent within cytokines producing memory CD4+, CD8+, and γδ T cell subtypes, indicating that in the **RO null** group, the memory T cell differentiation was not affected by the absence of CD45RO expression. Furthermore, we noticed that the average frequency of CD45RA+ T cells in the **RO+** cattle was similar to those in the **RO null**, which suggests that CD45RA expression might not depend on CD45RO. In the **RO+** cattle, only less than 20% of CD45RO+ CD4+ T cells produced IFNγ, which means that more than 80% of CD45RO+ cells did not produce effector cytokine upon in vitro stimulation. These experimental observations strongly suggest that using CD45RO as a marker for memory T cells in cattle might be overestimating and even misleading.

The classical paradigm assumes that switching of CD45 isoforms from CD45RA to CD45RO is an essential process associated with the memory T cell differentiation [7,8,53,75,76,77]. However, the data from the **RO null** cattle suggest that, at least in a fraction of cattle, switching of CD45 isoforms is not required for the induction of memory T cells. We noticed that the average percentage of CD45RA+ lymphocytes in the **RO null** cattle was similar to that in **RO+** cattle. Further, the average frequencies of CD45RA+ cells within CD4+ and CD8+ T cell subtypes in the **RO+** cattle were also close to those in the **RO null**. The evidence suggests that the isoform switch might not be necessary to induce memory T cell population in all cattle. Therefore, the relevance of CD45RO as a signature marker for memory bovine T cells is questionable. 

Our data demonstrate that CD45RA/RO expression pattern differs in distinct bovine T cell subtypes. While the proportion of CD45RA+ cells in the total lymphocytes was always higher than that of CD45RO+ cells, the pattern varied significantly within the distinct bovine T cell subtypes. In γδ (more than 90%) and CD4+ (60%), CD45RO expression was dominant; however, in CD8+ T cells, CD45RO expression was relatively low (about 30%) but CD45RA expression was significantly high. These data indicate a distinct pattern of CD45RA/RO clustering in bovine T cell subtypes. The tendency of our data is in partial agreement with several previous reports, where γδ T cells were extensively CD45RO+, CD4+ T cells were predominantly CD45RO+, but CD8+ T cells were mostly CD45RO- [8,23,44]. Interestingly, this pattern has been similarly reported in human T cell subtypes [78,79,80,81,82]. Therefore, the distinct pattern of isomer clustering reflects that the CD45RA/RO expression is strongly associated with bovine T cell subtypes. 

We noticed that even within the cytokine producing T cell population, the CD45RA/RO expression pattern was associated with distinct T cell subtypes, which is in agreement with several previous reports. It has been reported that the antigen-specific memory CD4+ population is within the CD45RO+ population [5,8,23,24,38,39], but that of CD8+ T cells contains both CD45RA+ and CD45RO+ fractions [8,34,35,37]. Previously, cytokine-producing ovalbumin (OVA)-specific memory bovine CD4+ T cells were found within the CD45RO+ sub-population, which is in line with another report, where the CD45RO+ fraction of the CD4+ T cells dominantly produced IFN-γ and IL4 in both blood and lymph nodes [8,23]. In contrast, a different pattern has been reported in the context of bovine CD8+ T cells. While the *S. uberis* and *BCG*-specific memory CD8+ T cells were mostly found within CD45RO+ population, those specific to *Theileria parva* were under both CD45RA+ and CD45RO+ fractions [8,34,35,37]. Our analysis suggests that the memory bovine T cells could be both CD45RO+ and CD45RA+ with the dominant expression of CD45RO in the CD4+ T cells but CD45RA in the CD8+ subtype. Therefore, the association between CD45RA/RO expression and bovine T cell subtypes was also reflected within IFNγ and IL-4 inducing cells.

As CD45RA/RO expression on T cells is more related to distinct subtypes than the memory phenotype, we speculate that their expression pattern could be associated with T cell subtype-specific functions, which has been reported in humans [72,83,84,85]. For instance, *Dengue virus (DENV)* specific CD45RA+ CD4+ T cells, *Epstein-Barr virus* (EBV) specific CD45RA+ CD8+ T cell, and *M tuberculosis* as well as *cytomegalovirus* (CMV) specific CD8+ γδ T cells expressing CD45RA demonstrated cytotoxic activity [72,83,84,85]. Additionally, CD45RA+ but not CD45RA- CD8+ T cells isolated from *HIV-1* infected patients selectively demonstrated cytotoxicity under in vitro assay, indicating that killing ability could be related to CD45RA expression on these cells [74]. In contrast, CD45RO expression in the CD4+ T cells could be more related to immunoregulatory functions such as helping B cells to produce immunoglobulins. CD45RO+ CD4+ T cells showed an improved ability to adhere with immortalized B cell lines than the CD45RA+ cells [86]. Furthermore, CD45RO+ CD4+ T cell isolated from the blood of healthy humans stimulated B cells to produce high levels of IgM and IgG in vitro, which were drastically reduced when CD45RO+ CD4+ T cells were removed from the culture [11,87]. These lines of evidence provide a new perspective for interpreting the distinct pattern of CD45RA/RO clustering on T cells that their expression could be related to the distinct T cell subtypes and their specific effector functions. Therefore, more research is required to appropriately understand how distinct CD45RA/RO expression is related to the cell-specific functions in cattle. Nevertheless, more biomarkers are warranted to precisely identify the memory bovine T cell population in cattle. 

## 5. Conclusions

Our data contrast the classical CD45RA/RO paradigm and suggest that CD45RO expression on CD45RA- T cells is not strongly correlated with memory identification. Rather than with memory phenotype, the pattern of CD45RA/RO distribution on the T cells is associated more with distinct T cell subtypes. Therefore, future research should target to identify novel markers for memory T cell population and also define the function of CD45RA/RO isoforms in cattle.

## Figures and Tables

**Table 1:** Primary antibodies.

Specificity	Clone	Isotype	Source
bCD3	MM1A	IgG1	WSUMAC
bCD4	CC8	IgG2a	Bio-Rad
bCD25	LCTB2A	IgG3	WSUMAC
bTCRδ	GB21A	IgG2b	WSUMAC
bTCRδ	CACT61A	IgM	WSUMAC
bCD45RA	GC6A	IgM	WSUMAC
bCD45RO	ILA116	IgG3	WSUMAC
bCD62L	BAQ92A	IgG1	WSUMAC
bIFNγ	CC302	IgG1	Bio-Rad
bIL4	CC303	IgG2a	Bio-Rad
**Specificity**	**Clone**	**Conjugated** **Fluorescence**	**Source**
bCD4	CC8	PE	Bio-Rad
bCD8	CC63	FITC	Bio-Rad
bCD8	CC58	PE	Bio-Rad
bCD25	IL-A111	PE	Bio-Rad
bCD25	IL-A111	FITC	Bio-Rad
bCD62L	CC32	FITC	Bio-Rad
bIFNγ	CC302	PE	Bio-Rad

b, Bovine. WSUMAC, Washington State University Monoclonal Antibody Center. Bio-Rad, Bio-Rad Laboratories, Inc. Hercules, CA, USA. Note: All the primary antibodies are monoclonal and generated from mice.

**Table 2:** Secondary antibodies and isotype controls.

Specificity	Secondary Antibodies	Source
IgG1	Anti-mouse IgG1-APC	BioLegend
IgG1	Anti-mouse IgG1-Biotin	BioLegend
IgG2a	Anti-mouse IgG2a-APC	BioLegend
IgG2b	Anti-mouse IgG2b-PE	BioLegend
IgG2b	Anti-mouse IgG2b-Biotin	BioLegend
IgG2b	Anti-mouse IgG2b-FITC	BioLegend
IgG3	Anti-mouse IgG3-Biotin	BioLegend
IgM	Anti-mouse IgM-BV421	BioLegend
Biotin	Streptavidin Per-CP	BioLegend
**Isotype Controls**		**Source**
Mouse IgG1		BioLegend
Mouse IgG2a		BioLegend
Mouse IgG2b		BioLegend
Mouse IgG3		BioLegend
Mouse IgM		BioLegend
Mouse IgG1-FITC		BioLegend
Mouse IgG1-PE		BioLegend
Mouse IgG2a-FITC		BioLegend
Mouse IgG2a-PE		BioLegend

## Data Availability

Data are contained within the article and Supplemental File.

## Supplementary Materials

The following supporting information can be downloaded at: https://www.mdpi.com/article/10.3390/cells11111844/s1, Figure S1: CD45RO is not expressed by lymphocytes in some cattle; Figure S2: CD45RO and CD45RA are expressed differentially in T cell subtypes; Figure S3: Activation status varies between CD45RO+ and CD45RA+ subpopulations in CD4+, but not in CD8+ T cells; Figure S4: Expression of IFNg and IL-4 is not associated with CD45RO in CD4+ T cells; Figure S5: Expression of IFNg is not associated with CD45RO in CD8+ T cells; Figure S6: Expression of IFNg and IL4 is not associated with CD45RO in GD T cells.
