# Supplementary material for: Differential Expression of CD45RO and CD45RA in Bovine T Cells
Source: Cells. 2022 Jun 4;11(11):1844. doi: 10.3390/cells11111844 (PMC9180881; doi:10.3390/cells11111844)
Supplement: Supplementary file 1 [file cells-11-01844-s001.zip › cells-1717062-supplementary.pdf]

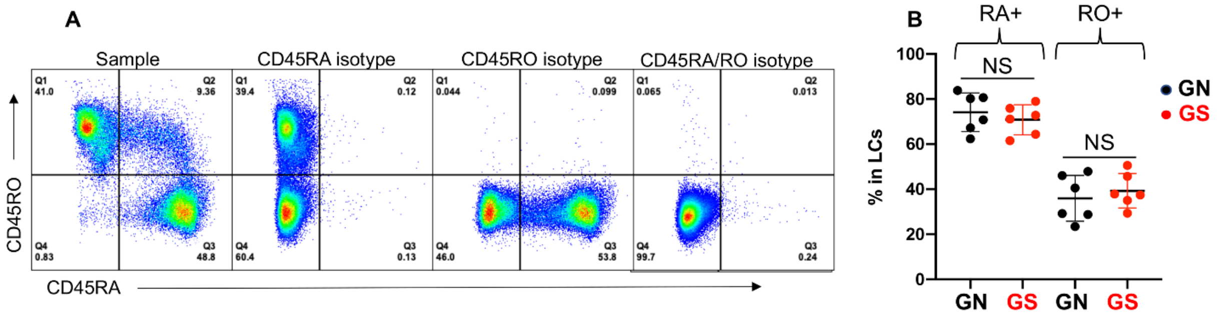

Supplementary Figure S1. CD45RO is not expressed by lymphocytes in some cattle. Lymphocytes were gated in PBMCs, and examined for expression of CD45RO and CD45RA. (A) Representative dot plots of samples and CD45RA or CD45RO isotype controls in lymphocytes. (B) Comparison of CD45RO (RO+) and CD45RA (RA+) expression in lymphocytes from Grain-fed (GN) and Grass-fed (GS) cattle. Data were the same as in Fig.1, and reanalyzed by one-way ANOVA with Tukey's Multiple Comparisons Test. Asterisks indicate statistical significance. NS: not significant.

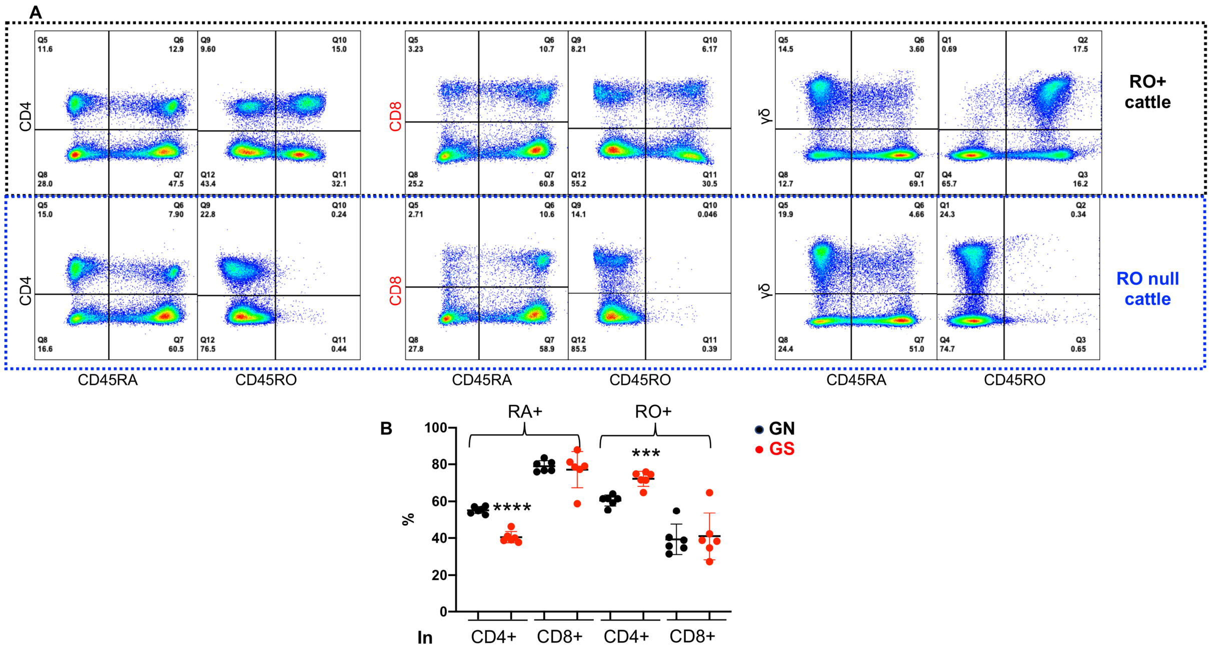

Supplementary Figure S2. CD45RO and CD45RA are expressed differentially in T cell subtypes. Lymphocytes were gated in PBMC, and examined for the expression of CD45RO and CD45RA. (A) Representative dot plots of samples and CD45RA or CD45RO isotype controls in CD4+, CD8 and γδ T cells in both CD45-expressing and CD45RO null cattle. (B) Comparison of CD45RO and CD45RA expression in CD4+ and CD8+ T cells from Grain-fed (GN) and Grass-fed (GS) cattle. Data were the same as in Fig.1, and reanalyzed by one-way ANOVA with Tukey's Multiple Comparisons Test. Asterisks indicate statistical significance. ***P < 0.001, ****p < 0.0001. NS: not significant.

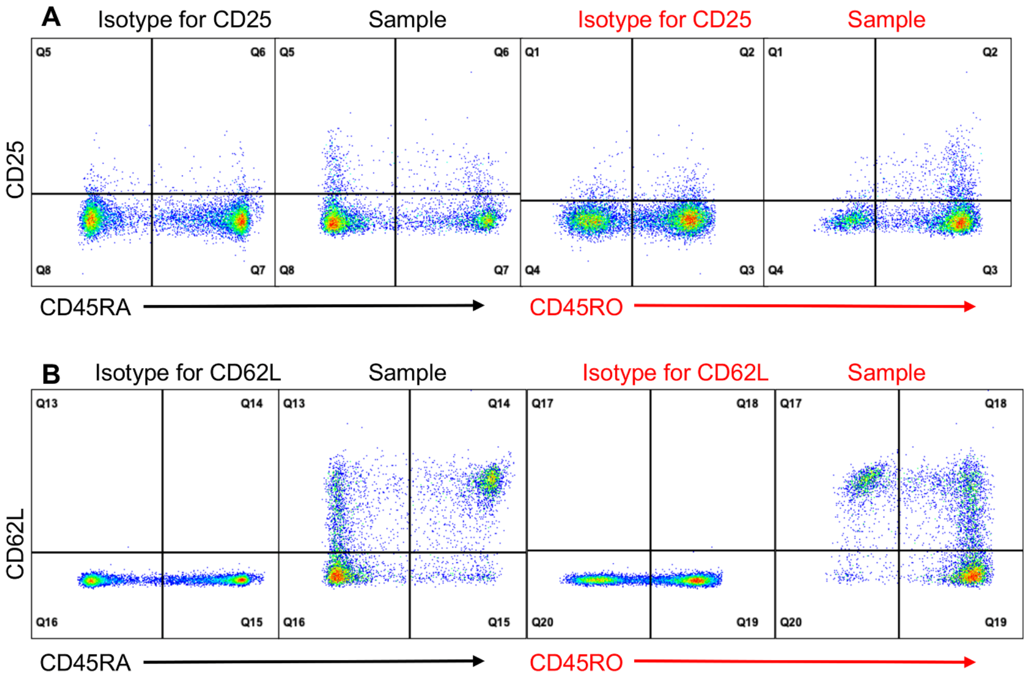

Supplementary Figure S3. Activation status varies between CD45RO+ and CD45RA+ subpopulations in CD4+, but not in CD8+ T cells. Lymphocytes were gated on CD4+ or CD8+ T cells in PBMCs, and examined for the expression of CD25 and CD62L in either RO+ or RA+ subpopulations. (A-B) Representative dot plots of samples and isotypes for CD25 (A) or CD62L (B) in RA+ or RO+ gated on CD4+ T cells.

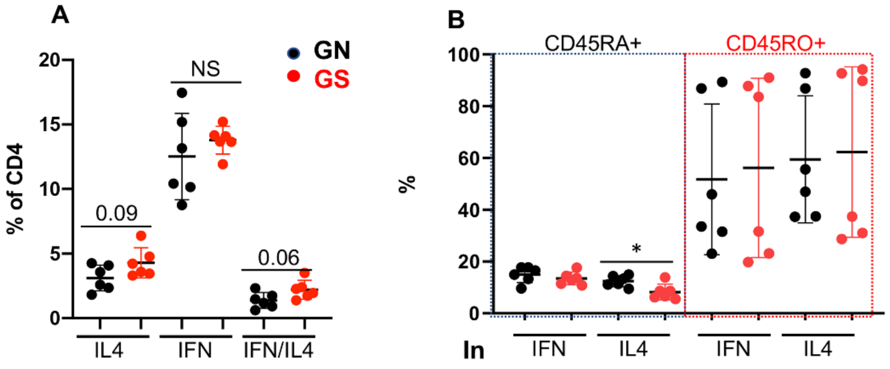

Supplementary Figure S4. Expression of IFNγ and IL-4 is not associated with CD45RO in CD4+ T cells. Purified PBMCs were stimulated with activation cocktail for 4 hours before intracellular staining for IFNγ and IL4, plus surface staining with antibodies to identify CD4 T cells, and their expression of CD45RO and CD45RA. (A) Comparison of IFNγ+ and IL4+ in CD4+ T cells from Grain-fed (GN) and Grass-fed (GS) cattle. (B) Comparison of CD45RO+ or CD45RA+ in IFNγ+ or IL4+ CD4+ T cells from GN and GS cattle. Data were the same as in Fig.4, and reanalyzed by one-way ANOVA with Tukey's Multiple Comparisons Test. Asterisks indicate statistical significance. *$P < 0.05$. NS: not significant.

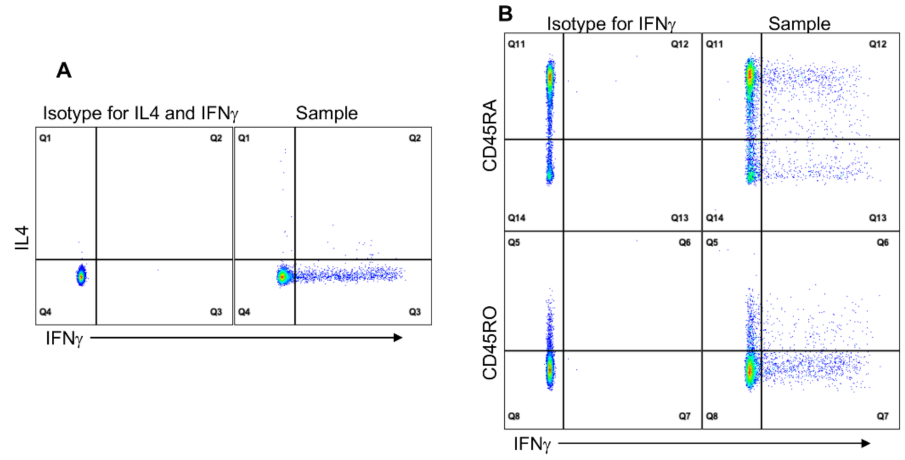

Supplementary Figure S5. Expression of IFNγ is not associated with CD45RO in CD8+ T cells. The same samples and treatment were applied as in Figure 4, but the analysis was gated on CD8+ T cells. (A) Representative dot plots of samples and isotypes for IFNγ and IL4 in CD8+ T cells. (B) Representative dot plots of samples and isotypes for IFNγ and CD45RA or CD45RO expressions on gated CD8+ T cells.

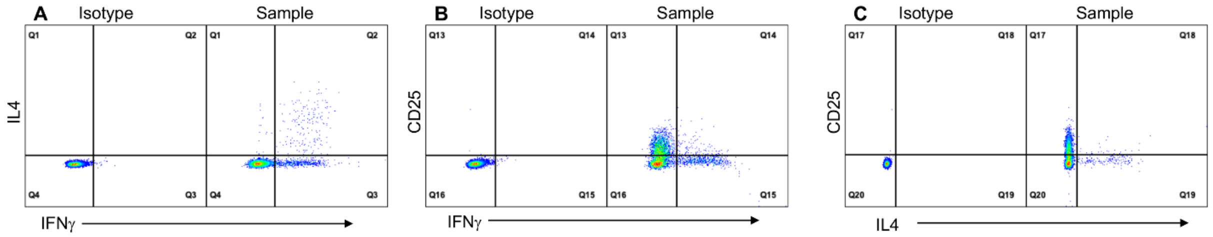

Supplementary Figure S6. Expression of IFNγ and IL4 is not associated with CD45RO in GD T cells. PBMCs were isolated from 12 cattle, which were tested for expression of CD45RO, CD62L, IFNγ and IL4 in a way similar to that in figures 4. (A) Representative dot plots of samples and isotypes for IFNγ and IL4 in γδ T cells. (B) Representative dot plots of samples and isotypes for IFNγ and CD25 in γδ T cells. (C) Representative dot plots of samples and isotypes for CD25 and IL4 in γδ T cells.
